# Comparison of emergency surgeries for obstructed colonic cancer with elective surgeries: A retrospective study

**DOI:** 10.12669/pjms.316.8277

**Published:** 2015

**Authors:** Ilker Sucullu, Yavuz Ozdemir, Mehmet Cuhadar, Ahmet Ziya Balta, Ergun Yucel, Ali Ilker Filiz, Bulent Gulec

**Affiliations:** 1 Ilker Sucullu, Associate Professor, Department of General Surgery, GATA Haydarpasa Training Hospital, Istanbul, Turkey; 2 Yavuz Ozdemir, Assistant Professor, Department of General Surgery, GATA Haydarpasa Training Hospital, Istanbul, Turkey; 3 Mehmet Cuhadar, Resident Doctor, Department of General Surgery, GATA Haydarpasa Training Hospital, Istanbul, Turkey; 4 Ahmet Ziya Balta, Associate Professor, Department of General Surgery, GATA Haydarpasa Training Hospital, Istanbul, Turkey; 5 Ergun Yucel, Associate Professor, Department of General Surgery, GATA Haydarpasa Training Hospital, Istanbul, Turkey; 6 Ali Ilker Filiz, Associate Professor, Department of General Surgery, GATA Haydarpasa Training Hospital, Istanbul, Turkey; 7 Bulent Gulec, Professor, Department of General Surgery, GATA Haydarpasa Training Hospital, Istanbul, Turkey

**Keywords:** Obstruction, Colon cancer, Morbidity, Mortality

## Abstract

**Objective:**

Colon cancer patients presented with obstruction were known to have worse postoperative morbidity and mortality rates, but conflicting data has been reported in recent years. We aimed to investigate postoperative complication rates, and short and long-term oncological outcomes in patients with colon cancer treated with either emergency surgery due to obstruction or elective surgery.

**Methods:**

Two hundred fifty two patients were analyzed. Patients presented with obstruction and underwent an emergency surgery, and patients operated under elective circumstances were compared according to their demographic variables, tumor characteristics, and short and long term treatment outcomes.

**Results:**

Distribution of age, gender and comorbidities were similar between both the groups. Need for an end colostomy was significantly higher in obstructed patients (22.7% vs 1.6%, respectively). Obstructed patients were tending to be at an advanced stage. Postoperative morbidity and mortality, and prognosis of colon cancer patients presented with obstruction is worse than patients operated under elective circumstances.

**Conclusions:**

Colon cancer patients presented with obstruction constitutes more than one quarter of all patients. These patients have significantly higher morbidity and mortality rates. Obstructed colon cancer usually appears at advanced stage. Primary resection and anastomosis is safe in most of the cases.

## INTRODUCTION

Colon cancer is one of the most common cancers in the world.^1^,^2^ Obstruction is a common complication of the colon cancer and 8-29% of the cases present with obstruction.^3^,^4^ This situation is associated with a higher morbidity and mortality rates, and poor prognosis.^5^

Surgery is the only potentially curable treatment option in colonic cancer patients operated either in emergency or elective settings. But urgent surgical treatment of obstructed colon cancer is associated with higher complication and mortality rates despite advances in the surgical techniques and intensive care. Moreover, curative resection rates are lower in emergency operation settings.^6^

Development of a complete colonic obstruction requires a long period of time, and it tends to be at a more advanced stage at presentation.^7^ Overall survival rate after a potentially curative resection of an obstructed colon cancer is worse than non-obstructed cases.^8^

Hence, we undertook this study with the primary aim to compare the postoperative complication rates, and short and long-term oncological outcomes of our colonic cancer patients treated with either emergency surgery due to obstruction or elective surgery.

## METHODS

A retrospective review of all patients who underwent curative surgery for colonic malignancy from January 2004 to December 2013 was performed. A total of 538 patients were recorded in our database. Rectal cancer patients (n=186), patients who had a pathological diagnosis other than adenocarcinoma (n=4), patients who lost at follow up (n=71), and patients who had a palliative surgery without primary tumor resection (n=25) were excluded from the study. The remaining 252 patients were analyzed. Local ethics committee approval was obtained.

Diagnosis of obstruction was based on clinical, radiologic and operative findings, which include the symptoms of abdominal pain and constipation, signs of abdominal distension and abnormal bowel gaseous distension on plain radiographs, and operative findings of proximal bowel distension and edema. Prior to surgery, fluid resuscitation, parenteral antibiotics, optimization of their medical conditions, and nasogastric decompression was administered to every patient presented with obstruction.

Patients were stratified into two groups. Group 1 consisted of patients who underwent surgery for obstructed colonic cancer under emergency situations, and Group 2 consisted of patients operated electively for colonic cancer.

A radical resection of the colonic tumor along with high ligation of the vascular pedicle and accompanying lymphatic drainage was performed in all cases. Hartmann’s procedure, ileostomy, or colostomy were used in high-risk patients, and in the presence of fecal peritonitis or septic shock. Surgery was assumed to be curative in case of no evidence of macroscopic residual tumor once resection had been completed and reported tumor free resection margins of the specimen.

Postoperative ileus was defined as the absence of bowel movements or passage of flatus at least 120 hours.^9^ Anastomotic leak was defined as a disruption of the anastomosis identified at reoperation or extravasation of contrast medium at the anastomotic site on an imaging study, irrespective of the presence of symptoms.^10^ Death resulted from any cause at the postoperative first month was considered as mortality.

### Statistical Analysis

All statistical analyses were performed using SPSS 16.0 (SPSS Inc., Chicago, IL, USA). Student’s t test or Mann Whitney U test and Chi-squared test were used in order to determine the distribution of demographic characteristics of patients and to make probability charts. The Kaplan-Meier method was used in order to calculate cumulative survival rates according to the groups. The difference in survival rates between groups was calculated by using the log-rank test. Cox regression analysis was performed in order to determine prognostic factors that affected the survival time (p<0.05).

## RESULTS

A total of 252 patients who underwent surgery for colonic cancer were included into the study. While 186 (73.8%) of these patients underwent an elective surgery, 66 (26.2%) patients presented with an obstruction and underwent an emergency surgery. Demographic characteristics of the patients are summarized in Table-I.

**Table-I:** Demographic characteristics of the patients.

		Obstructed (n=66)	Non-obstructed (n=186)	P value
Mean age (year)		71.9±14.4	71.4±10.6	0.300
Gender	Male	33	114	0.740
	Female	33	72	
Comorbidity	Cardiovascular	14	41	0.174
	Diabetes Mellitus	11	39	0.288
	Respiratory	6	15	0.486
	Renal	4	19	0.229
	Hypertension	36	89	0.214

The most common surgical procedure was anterior resection in non-obstructed group (42.5%) and right hemicolectomy in the obstructed group (33.3%). Surgical procedures, status of adjacent tissue invasion and liver metastasis, complications related to tumor obstruction, and need for additional tissue resection or diversion are shown in Table-II.

**Table-II:** Operation details.

		Obstructed (n=66)	Non-obstructed (n=186)	P value
Surgical Procedure	Anterior resection	9	79	<0.001
	Left hemicolectomy	10	29	
	Transvers colectomy	4	4	
	Right hemicolectomy	22	60	
	Total colectomy	6	10	
	Total proctocolectomy	0	1	
	Hartmann procedure	15	3	
Findings in the exploration	Peritoneal invasion	7	21	0.542
	Uterine/ovarian invasion	4	8	0.388
	Bladder invasion	0	5	0.216
	Tumor perforation	10	5	0.001
	Fistula formation (enterocutaneous/enteroenteral/enterovesikal)	2	4	0.544
	Liver metastasis	11	17	0.078
Additional surgical tissue resections	Small bowel resection	3	13	0.357
	Abdominal wall resection	1	4	0.608
	Bladder resection	0	4	0.294
	Hysterectomy	1	3	0.719
	Liver resection	3	12	0.414
	Splenectomy	3	3	0.267
Diversion		1	5	0.504

Tumor was located in the sigmoid colon in almost half of the cases in the non-obstructed group (45.7%). This ratio was 39.4% in the obstructed group. While need for an end colostomy with sigmoid resection was only 1.6% in non-obstructed group, this ratio was increased to 22.7% in obstructed patients. There was only one patient with T2 tumor in the obstructed group, and the remaining lesions were T3 and T4 (53 and 12, respectively). Lymph node involvement and angiolymphatic invasion rates were higher in the obstructed group. Tumor characteristics are summarized in Table-III.

**Table-III:** Tumor characteristics.

		Obstructed (n=66)	Non-obstructed (n=186)	P value
Primary tumor localization	Sigmoid	26	85	0.818
	Descending colon	13	24	
	Transvers colon	10	11	
	Ascending colon	17	65	
	Synchronous tumor	0	1	
Differentiation	Well	7	38	0.340
	Moderate	51	127	
	Bad	2	12	
	Not reported	6	9	
Synchronous polyp in the specimen		9	37	0.173
Angiolymphatic invasion		31	52	0.004
Total number of harvested lymph nodes		11.9±9.4	14.3±9.8	0.010
Total number of metastatic lymph nodes		1.8±3.1	1.3±2.7	0.006
pT	1	0	17	<0.001
	2	1	24	
	3	53	124	
	4	12	21	
pN	0	24	114	0.002
	1	34	51	
	2	8	21	
pM	0	51	163	0.037
	1	15	23	
Stage	1	0	33	<0.001
	2	21	74	
	3	30	56	
	4	15		23

The rate of postoperative mortality was significantly higher in the obstructed group (21.2% vs 4.8%, p=0.003). There was also a significant difference between obstructed and non-obstructed groups according to postoperative cardiac (21.2% vs 5.9%, respectively; p=0.001), and respiratory (28.8% vs 5.9%, respectively; p<0.001) complications, surgical site infection (42.4% vs 24.2%, respectively; p=0.005), and abscess formation (15.2% vs 3.8%, respectively; p=0.003). Rehospitalization rates were 7.6% in the obstructed group and 5.4% in the non-obstructed group (p=0.135). Postoperative morbidity and mortality rates are shown in Table-IV.

**Table-IV:** Postoperative morbidity and mortality.

		Obstructed (n=66)	Non-obstructed (n=186)	P value
Postoperative Complications	Cardiac	14	11	0.001
	Respiratory	19	11	<0.001
	Deep vein thrombosis	0	1	0.738
	Neurological	6	5	0.039
	Sepsis	11	19	0.123
	Surgical site infection	28	45	0.005
	Wound dehiscence	4	5	0.185
	Postoperative ileus	5	5	0.088
	Abscess	10	7	0.003
	Fistula formation	4	5	0.185
	Urinary tract infection	6	12	0.320
	Anastomosis dehiscence	6	9	0.169
Reoperation		11	19	0.123
Postoperative mortality (30 day)		14	9	0.003
Rehospitalization		5	10	0.351

**Fig.1:**
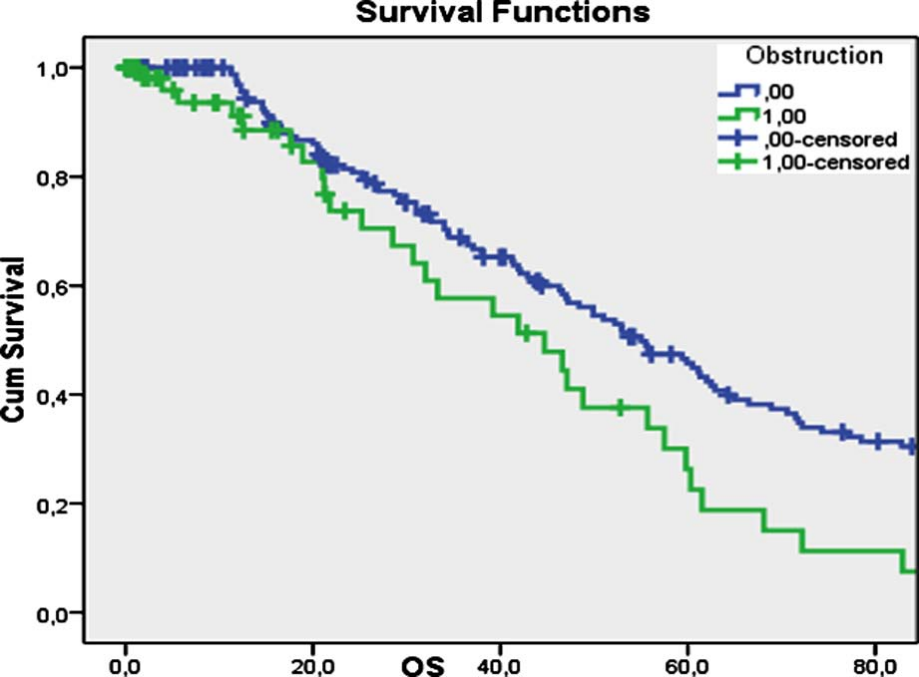
Kaplan Meier Curve of overall survival for obstructed or non-obstructed colonic tumor patients.

Overall survival was significantly shorter in patients with an obstructed colonic tumor (p=0.016). Kaplan-Meier curves representing overall survival for the presence of tumor obstruction is shown in Fig.1.

## DISCUSSION

Despite the technical improvements in the diagnosis and treatment of colonic cancer and the increased awareness, up to 30% of the patients presented with colonic obstruction.^3^,^11^ These patients have increased postoperative morbidity and mortality rates according to existing literature.^3^,^12^,^13^ Increased intraabdominal pressure, electrolyte imbalance, dehydration, and increased risk of fecal contamination are some of the contributing factors effecting postoperative morbidity and mortality.^14^,^15^ In our study, patients presented with obstruction constituted 26.2% of colonic cancer patients. Our postoperative morbidity and mortality rates were significantly higher in obstructed group.

Right hemicolectomy with ileocolic anastomosis is the preferred surgical procedure for obstructed right colon tumors.^15^ Despite the significant changes in tackling left sided colonic obstruction, its treatment still remains controversial. Some compounding factors like age, advanced disease, systemic sepsis, and comorbidities must be taken into account while choosing appropriate procedure.^16^ Avoidance of complications of a stoma, the risk of a second operation, and a better quality of life are some of the advantages of a one-stage resection and anastomosis.^17^ Sumise et al. evaluated one-staged resection and anastomosis in patients with obstructed colorectal cancer according to their age. They reported similar postoperative mortality and morbidity rates in these groups.^18^

Presence of associated proximal colon damage or a synchronous tumor can be treated with a subtotal colectomy. Hartmann procedure is usually chosen in patients with perforation and peritoneal seeding, or in severely ill patients. However rate of stoma creation is still high in left-sided colonic obstruction, because of higher rates of anastomotic complications in these patients. In our study, Hartmann procedure was needed significantly more in patients with obstructed colon cancer (22.7% vs 1.6%), but our diversion rates were similar between 2 groups.

Tan K and Sin R investigated the factors predicting postoperative morbidity in colorectal cancer patients presented with obstruction in an Asian population.^19^ Patients who had a higher American Society of Anesthesiologists (ASA) score, patients older than 60 years old, and patients who had preoperative renal impairment have worse complications. Tekkis et al. evaluated early outcomes after surgical treatment of malignant large bowel obstruction.^20^ Old age, higher grade of ASA score, advanced Dukes stage, and the emergency surgery were identified as risk factors affecting operative mortality. In our study, respiratory and neurological complications, surgical site infections, and abscess formation were seen significantly high in obstructed patients. Also postoperative mortality rates were significantly high in obstructed colon cancer patients.

Most of the previous studies have reported that obstructed colonic cancers present at a more advanced stage.^12^ Tumor size does not reflect the stage of the tumor, and there is no relationship between the size of the tumor and the presence of obstruction.^21^ Ratto et al. reported that bowel obstruction was the worst predictive factor for prognosis.^22^ Mulcahy et al. reported the factors like the presence of perforation, advanced tumor, poor differentiation, mucin production, and the presence of vascular and neural invasion as negative prognostic factors.^5^ Beside these factors, inflammatory reaction to the tumor may influence the prognosis.^23^ In this present study, patients with obstructed colon cancer had more advanced stage. But the distribution of tumor differentiation was similar between groups. Rate of presence of angiolymphatic invasion was significantly higher in obstructed group.

The rate of synchronous neoplasia including adenomas and adenocarcinomas was reported to be as high as 26.8%, in a series of colectomies and intraoperative colonoscopy for malignant obstruction.^24^ Pathologic evaluation of our series revealed synchronous adenomatous polyps in 9 patients (13.6%) with obstructed tumor and 37 patients (19.9%) with non-obstructed tumor. There was only one synchronous malign tumor in non-obstructed group. There was no difference between groups for both kinds of lesions.

Patients presented with obstruction have a worse prognosis according to existing literature.^3^,^12^,^13^ Progression of tumoral obstruction is a result of carcinogenesis and may take several years. During this period hematogenous and lymphogenous metastasis is more likely to occur and reported data shows advanced disease in obstructed patients.^25^ In our study, patients in the obstructed group were likely to have advanced disease and have poor prognosis when compared with non-obstructed group.

In conclusion, more than one quarter of colon cancer patients presented with obstruction, and these patients have significantly higher morbidity and mortality rates. Ratio of advanced stage of colon cancer was found higher in patients who presented with obstruction. Nevertheless, we have to perform the most adequate treatment with radical oncologic principles. One stage resection (primary resection and anastomosis) may be safe in most of the cases. However it is obvious that stoma will be needed in case of a fecal contamination or poor physical conditions.
